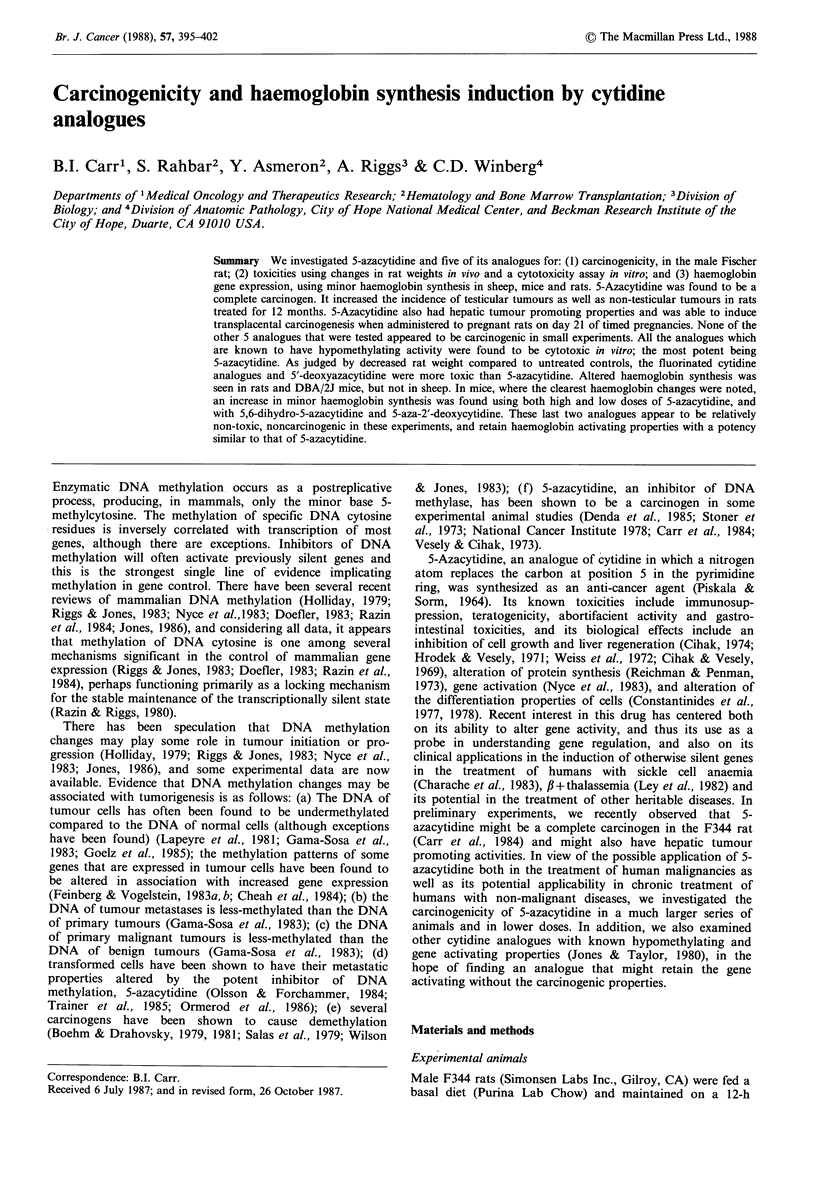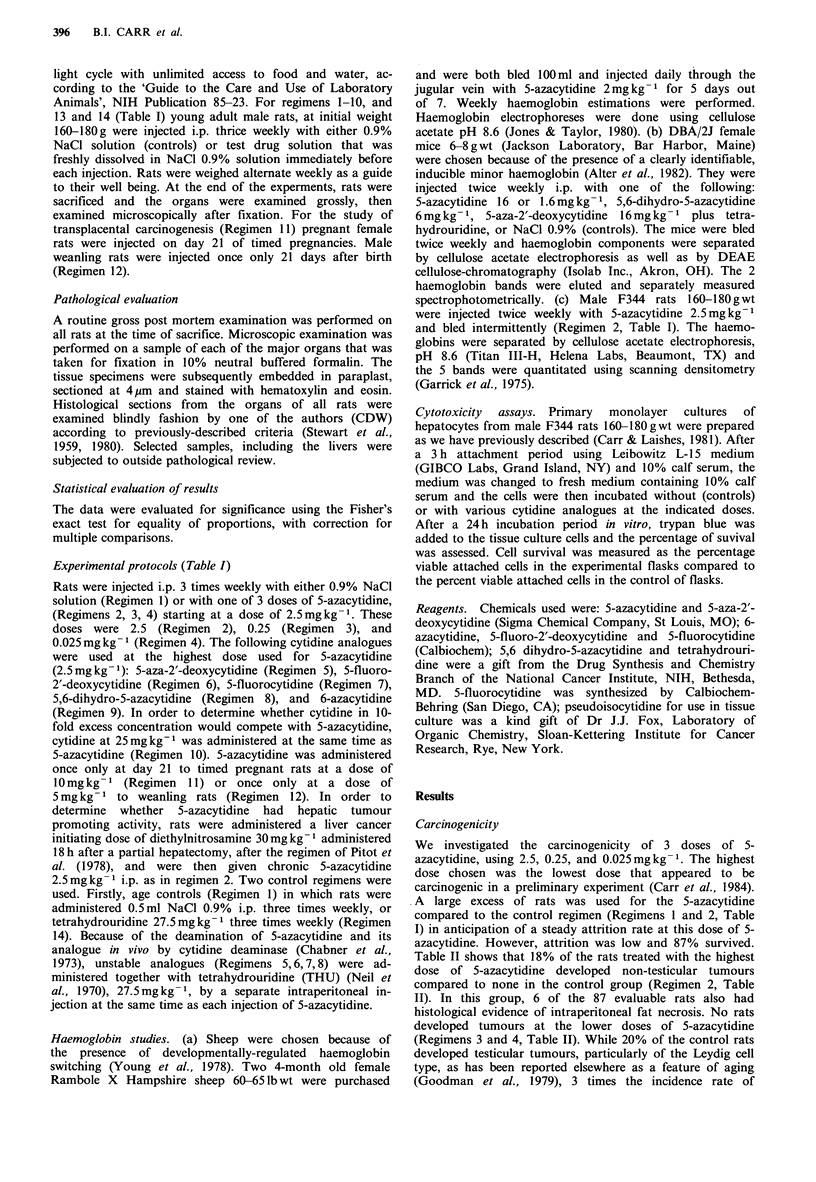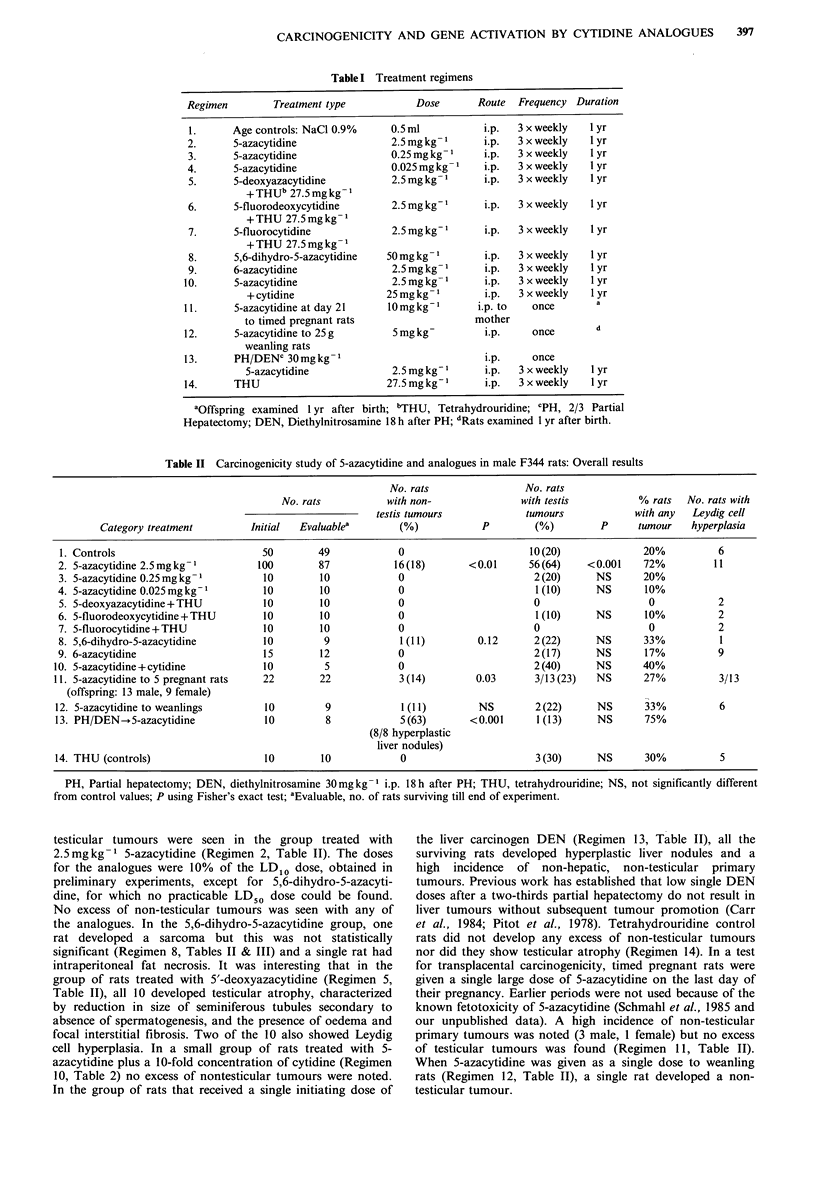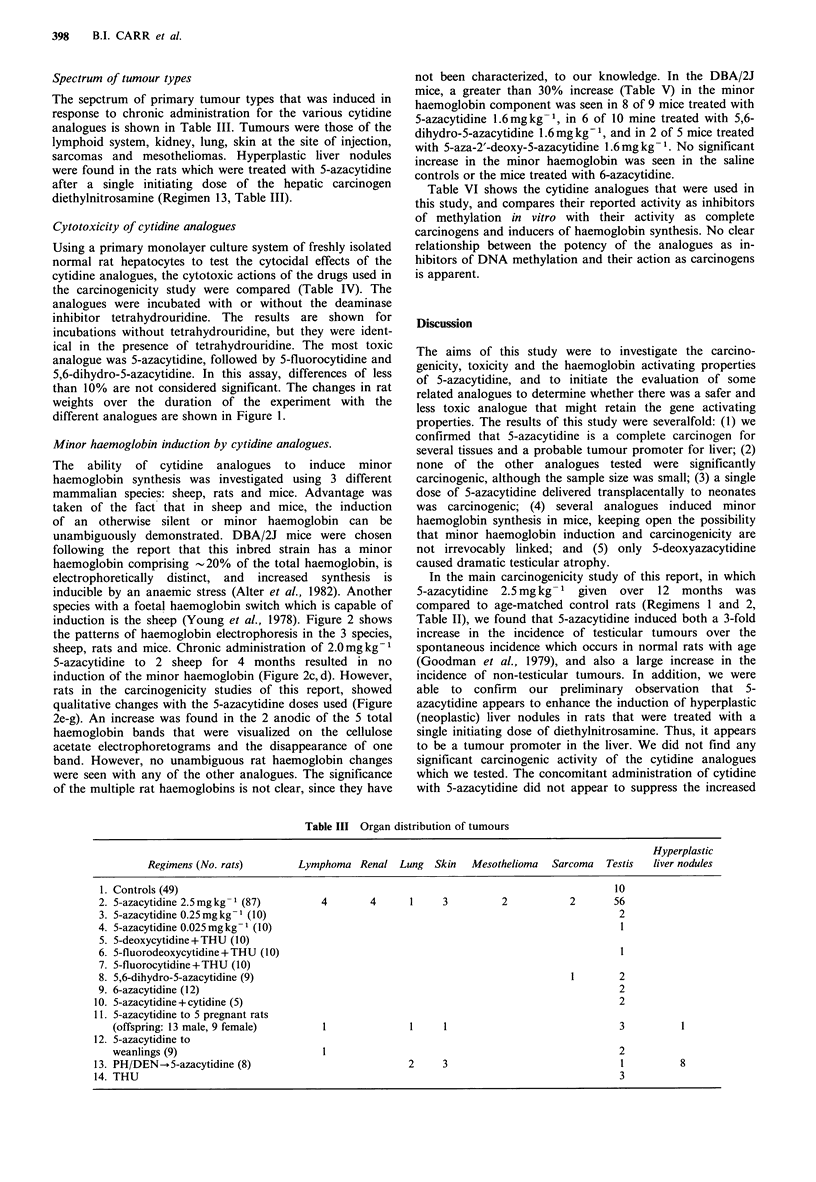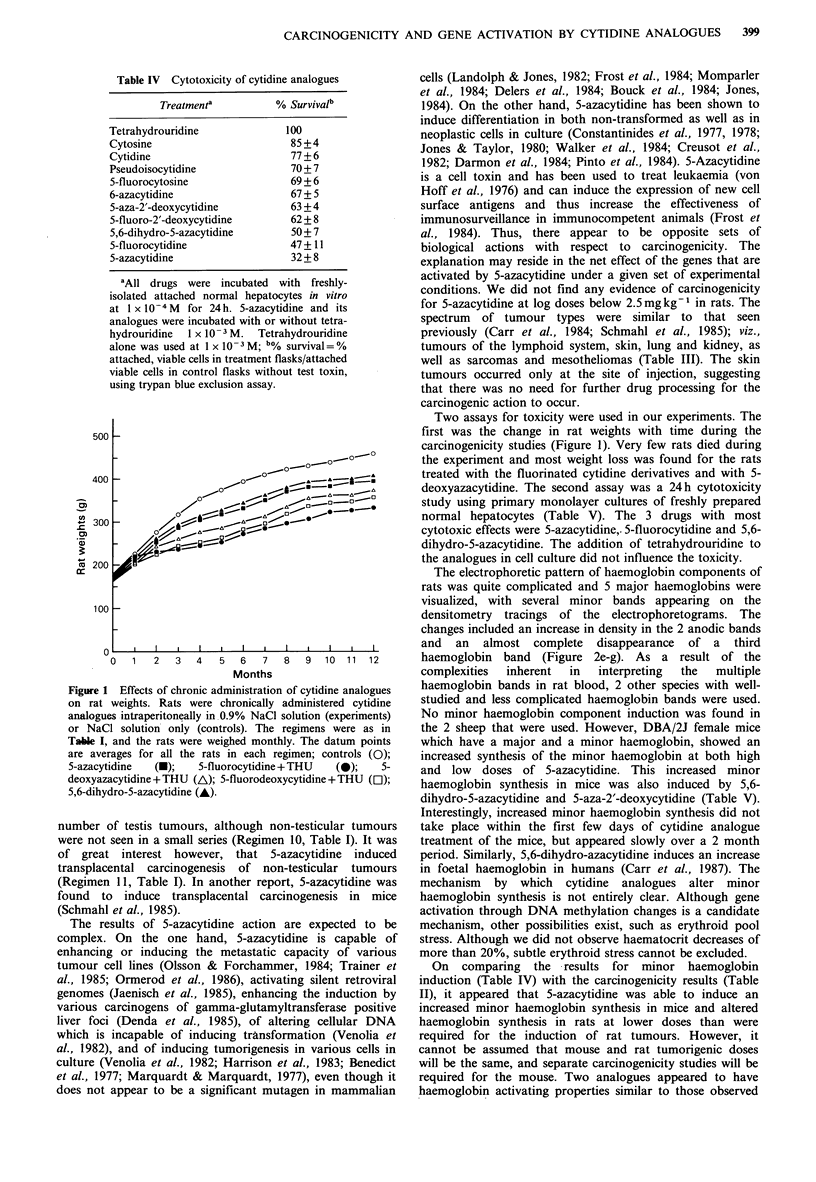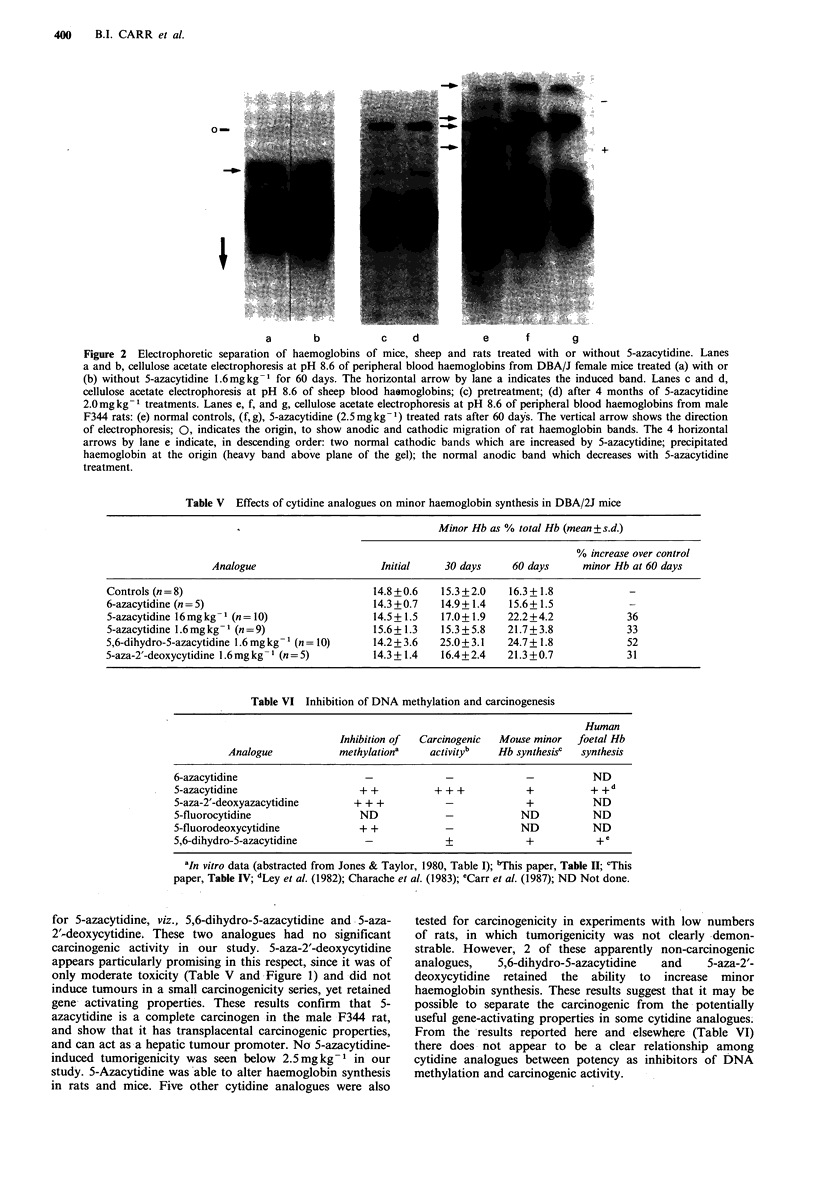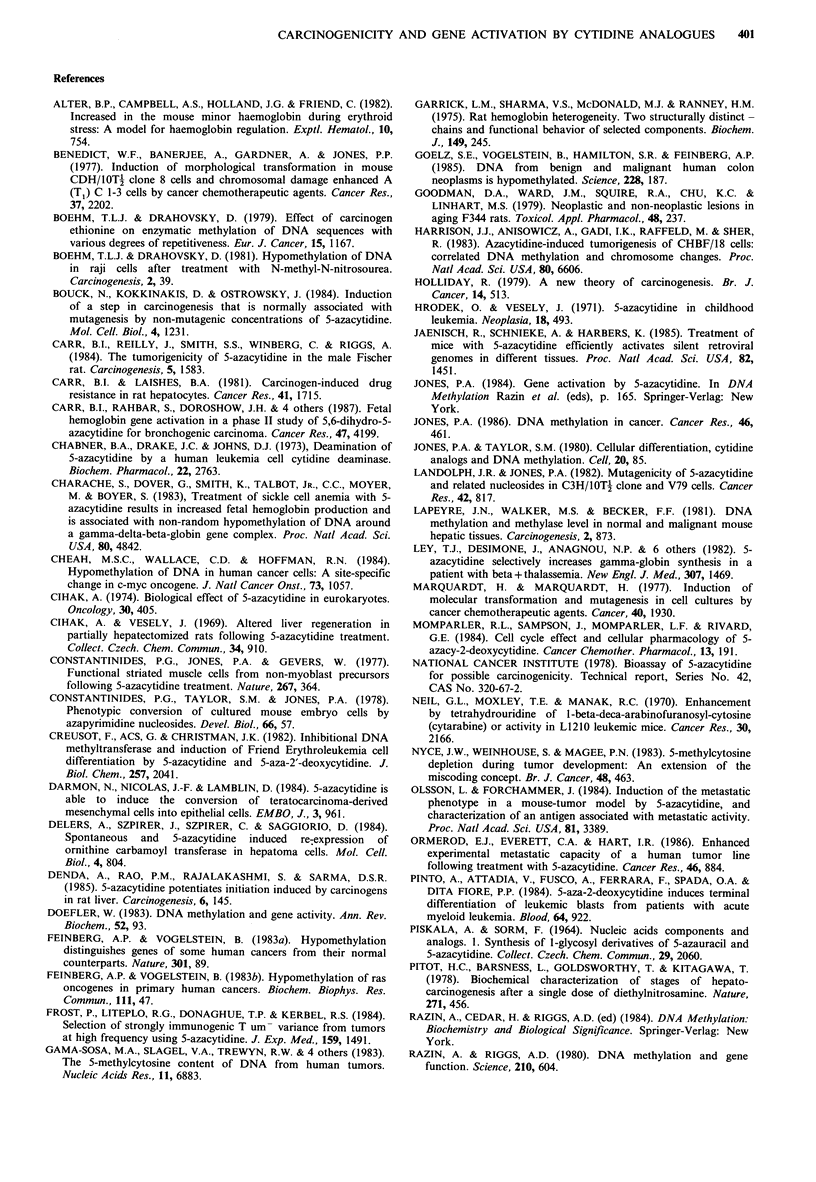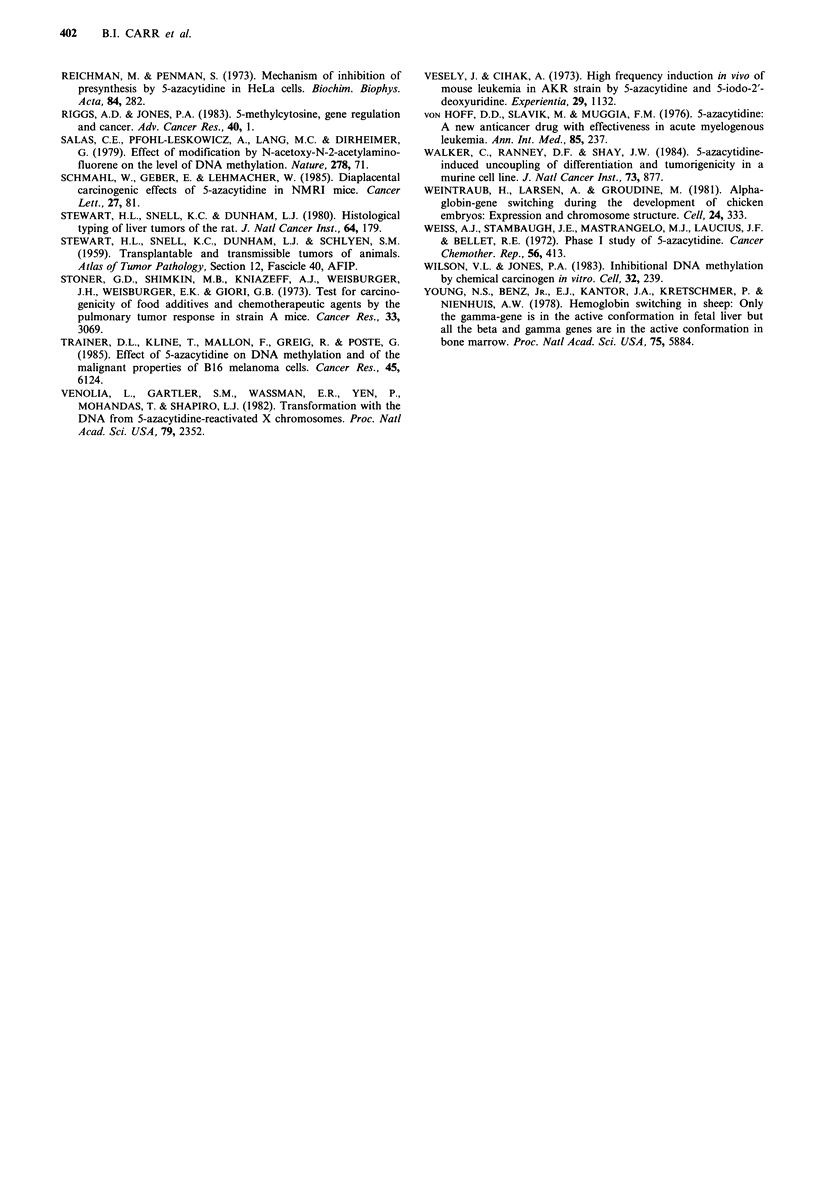# Carcinogenicity and haemoglobin synthesis induction by cytidine analogues.

**DOI:** 10.1038/bjc.1988.89

**Published:** 1988-04

**Authors:** B. I. Carr, S. Rahbar, Y. Asmeron, A. Riggs, C. D. Winberg

**Affiliations:** Department of Medical Oncology and Therapeutics Research, City of Hope National Medical Center, Duarte, CA.

## Abstract

**Images:**


Br. J. Cancer (1988), 57, 395–402
© The Macmillan Press Ltd., 1988

### Carcinogenicity and haemoglobin synthesis induction by cytidine analogues

B.I. Carr[1], S. Rahbar[2], Y. Asmeron[2], A. Riggs[3] & C.D. Winberg[4]

*Departments of [1]Medical Oncology and Therapeutics Research; [2]Hematology and Bone Marrow Transplantation; [3]Division of Biology; and [4]Division of Anatomic Pathology, City of Hope National Medical Center, and Beckman Research Institute of the City of Hope, Duarte, CA 91010 USA.*

**Summary** We investigated 5-azacytidine and five of its analogues for: (1) carcinogenicity, in the male Fischer rat; (2) toxicities using changes in rat weights *in vivo* and a cytotoxicity assay *in vitro*; and (3) haemoglobin gene expression, using minor haemoglobin synthesis in sheep, mice and rats. 5-Azacytidine was found to be a complete carcinogen. It increased the incidence of testicular tumours as well as non-testicular tumours in rats treated for 12 months. 5-Azacytidine also had hepatic tumour promoting properties and was able to induce transplacental carcinogenesis when administered to pregnant rats on day 21 of timed pregnancies. None of the other 5 analogues that were tested appeared to be carcinogenic in small experiments. All the analogues which are known to have hypomethylating activity were found to be cytotoxic *in vitro*; the most potent being 5-azacytidine. As judged by decreased rat weight compared to untreated controls, the fluorinated cytidine analogues and 5′-deoxyazacytidine were more toxic than 5-azacytidine. Altered haemoglobin synthesis was seen in rats and DBA/2J mice, but not in sheep. In mice, where the clearest haemoglobin changes were noted, an increase in minor haemoglobin synthesis was found using both high and low doses of 5-azacytidine, and with 5,6-dihydro-5-azacytidine and 5-aza-2′-deoxycytidine. These last two analogues appear to be relatively non-toxic, noncarcinogenic in these experiments, and retain haemoglobin activating properties with a potency similar to that of 5-azacytidine.

Enzymatic DNA methylation occurs as a postreplicative process, producing, in mammals, only the minor base 5-methylcytosine. The methylation of specific DNA cytosine residues is inversely correlated with transcription of most genes, although there are exceptions. Inhibitors of DNA methylation will often activate previously silent genes and this is the strongest single line of evidence implicating methylation in gene control. There have been several recent reviews of mammalian DNA methylation (Holliday, 1979; Riggs & Jones, 1983; Nyce *et al.*,1983; Doefler, 1983; Razin *et al.*, 1984; Jones, 1986), and considering all data, it appears that methylation of DNA cytosine is one among several mechanisms significant in the control of mammalian gene expression (Riggs & Jones, 1983; Doefler, 1983; Razin *et al.*, 1984), perhaps functioning primarily as a locking mechanism for the stable maintenance of the transcriptionally silent state (Razin & Riggs, 1980).

There has been speculation that DNA methylation changes may play some role in tumour initiation or progression (Holliday, 1979; Riggs & Jones, 1983; Nyce *et al.*, 1983; Jones, 1986), and some experimental data are now available. Evidence that DNA methylation changes may be associated with tumorigenesis is as follows: (a) The DNA of tumour cells has often been found to be undermethylated compared to the DNA of normal cells (although exceptions have been found) (Lapeyre *et al.*, 1981; Gama-Sosa *et al.*, 1983; Goelz *et al.*, 1985); the methylation patterns of some genes that are expressed in tumour cells have been found to be altered in association with increased gene expression (Feinberg & Vogelstein, 1983*a,b*; Cheah *et al.*, 1984); (b) the DNA of tumour metastases is less-methylated than the DNA of primary tumours (Gama-Sosa *et al.*, 1983); (c) the DNA of primary malignant tumours is less-methylated than the DNA of benign tumours (Gama-Sosa *et al.*, 1983); (d) transformed cells have been shown to have their metastatic properties altered by the potent inhibitor of DNA methylation, 5-azacytidine (Olsson & Forchammer, 1984; Trainer *et al.*, 1985; Ormerod *et al.*, 1986); (e) several carcinogens have been shown to cause demethylation (Boehm & Drahovsky, 1979, 1981; Salas *et al.*, 1979; Wilson & Jones, 1983); (f) 5-azacytidine, an inhibitor of DNA methylase, has been shown to be a carcinogen in some experimental animal studies (Denda *et al.*, 1985; Stoner *et al.*, 1973; National Cancer Institute 1978; Carr *et al.*, 1984; Vesely & Cihak, 1973).

5-Azacytidine, an analogue of cytidine in which a nitrogen atom replaces the carbon at position 5 in the pyrimidine ring, was synthesized as an anti-cancer agent (Piskala & Sorm, 1964). Its known toxicities include immunosuppression, teratogenicity, abortifacient activity and gastro-intestinal toxicities, and its biological effects include an inhibition of cell growth and liver regeneration (Cihak, 1974; Hrodek & Vesely, 1971; Weiss *et al.*, 1972; Cihak & Vesely, 1969), alteration of protein synthesis (Reichman & Penman, 1973), gene activation (Nyce *et al.*, 1983), and alteration of the differentiation properties of cells (Constantinides *et al.*, 1977, 1978). Recent interest in this drug has centered both on its ability to alter gene activity, and thus its use as a probe in understanding gene regulation, and also on its clinical applications in the induction of otherwise silent genes in the treatment of humans with sickle cell anaemia (Charache *et al.*, 1983), $\beta$+thalassemia (Ley *et al.*, 1982) and its potential in the treatment of other heritable diseases. In preliminary experiments, we recently observed that 5-azacytidine might be a complete carcinogen in the F344 rat (Carr *et al.*, 1984) and might also have hepatic tumour promoting activities. In view of the possible application of 5-azacytidine both in the treatment of human malignancies as well as its potential applicability in chronic treatment of humans with non-malignant diseases, we investigated the carcinogenicity of 5-azacytidine in a much larger series of animals and in lower doses. In addition, we also examined other cytidine analogues with known hypomethylating and gene activating properties (Jones & Taylor, 1980), in the hope of finding an analogue that might retain the gene activating without the carcinogenic properties.

### Materials and methods

### *Experimental animals*

Male F344 rats (Simonsen Labs Inc., Gilroy, CA) were fed a basal diet (Purina Lab Chow) and maintained on a 12-h

Correspondence: B.I. Carr.
Received 6 July 1987; and in revised form, 26 October 1987.

light cycle with unlimited access to food and water, according to the 'Guide to the Care and Use of Laboratory Animals', NIH Publication 85–23. For regimens 1–10, and 13 and 14 (Table I) young adult male rats, at initial weight 160–180 g were injected i.p. thrice weekly with either 0.9% NaCl solution (controls) or test drug solution that was freshly dissolved in NaCl 0.9% solution immediately before each injection. Rats were weighed alternate weekly as a guide to their well being. At the end of the experments, rats were sacrificed and the organs were examined grossly, then examined microscopically after fixation. For the study of transplacental carcinogenesis (Regimen 11) pregnant female rats were injected on day 21 of timed pregnancies. Male weanling rats were injected once only 21 days after birth (Regimen 12).

*Pathological evaluation*

A routine gross post mortem examination was performed on all rats at the time of sacrifice. Microscopic examination was performed on a sample of each of the major organs that was taken for fixation in 10% neutral buffered formalin. The tissue specimens were subsequently embedded in paraplast, sectioned at 4 $\mu$m and stained with hematoxylin and eosin. Histological sections from the organs of all rats were examined blindly fashion by one of the authors (CDW) according to previously-described criteria (Stewart *et al.*, 1959, 1980). Selected samples, including the livers were subjected to outside pathological review.

*Statistical evaluation of results*

The data were evaluated for significance using the Fisher's exact test for equality of proportions, with correction for multiple comparisons.

*Experimental protocols* (*Table I*)

Rats were injected i.p. 3 times weekly with either 0.9% NaCl solution (Regimen 1) or with one of 3 doses of 5-azacytidine, (Regimens 2, 3, 4) starting at a dose of 2.5 mg kg$^{-1}$. These doses were 2.5 (Regimen 2), 0.25 (Regimen 3), and 0.025 mg kg$^{-1}$ (Regimen 4). The following cytidine analogues were used at the highest dose used for 5-azacytidine (2.5 mg kg$^{-1}$): 5-aza-2′-deoxycytidine (Regimen 5), 5-fluoro-2′-deoxycytidine (Regimen 6), 5-fluorocytidine (Regimen 7), 5,6-dihydro-5-azacytidine (Regimen 8), and 6-azacytidine (Regimen 9). In order to determine whether cytidine in 10-fold excess concentration would compete with 5-azacytidine, cytidine at 25 mg kg$^{-1}$ was administered at the same time as 5-azacytidine (Regimen 10). 5-azacytidine was administered once only at day 21 to timed pregnant rats at a dose of 10 mg kg$^{-1}$ (Regimen 11) or once only at a dose of 5 mg kg$^{-1}$ to weanling rats (Regimen 12). In order to determine whether 5-azacytidine had hepatic tumour promoting activity, rats were administered a liver cancer initiating dose of diethylnitrosamine 30 mg kg$^{-1}$ administered 18 h after a partial hepatectomy, after the regimen of Pitot *et al.* (1978), and were then given chronic 5-azacytidine 2.5 mg kg$^{-1}$ i.p. as in regimen 2. Two control regimens were used. Firstly, age controls (Regimen 1) in which rats were administered 0.5 ml NaCl 0.9% i.p. three times weekly, or tetrahydrouridine 27.5 mg kg$^{-1}$ three times weekly (Regimen 14). Because of the deamination of 5-azacytidine and its analogue *in vivo* by cytidine deaminase (Chabner *et al.*, 1973), unstable analogues (Regimens 5, 6, 7, 8) were administered together with tetrahydrouridine (THU) (Neil *et al.*, 1970), 27.5 mg kg$^{-1}$, by a separate intraperitoneal injection at the same time as each injection of 5-azacytidine.

*Haemoglobin studies.* (a) Sheep were chosen because of the presence of developmentally-regulated haemoglobin switching (Young *et al.*, 1978). Two 4-month old female Rambole X Hampshire sheep 60–65 lb wt were purchased and were both bled 100 ml and injected daily through the jugular vein with 5-azacytidine 2 mg kg$^{-1}$ for 5 days out of 7. Weekly haemoglobin estimations were performed. Haemoglobin electrophoreses were done using cellulose acetate pH 8.6 (Jones & Taylor, 1980). (b) DBA/2J female mice 6–8 g wt (Jackson Laboratory, Bar Harbor, Maine) were chosen because of the presence of a clearly identifiable, inducible minor haemoglobin (Alter *et al.*, 1982). They were injected twice weekly i.p. with one of the following: 5-azacytidine 16 or 1.6 mg kg$^{-1}$, 5,6-dihydro-5-azacytidine 6 mg kg$^{-1}$, 5-aza-2′-deoxycytidine 16 mg kg$^{-1}$ plus tetrahydrouridine, or NaCl 0.9% (controls). The mice were bled twice weekly and haemoglobin components were separated by cellulose acetate electrophoresis as well as by DEAE cellulose-chromatography (Isolab Inc., Akron, OH). The 2 haemoglobin bands were eluted and separately measured spectrophotometrically. (c) Male F344 rats 160–180 g wt were injected twice weekly with 5-azacytidine 2.5 mg kg$^{-1}$ and bled intermittently (Regimen 2, Table I). The haemoglobins were separated by cellulose acetate electrophoresis, pH 8.6 (Titan III-H, Helena Labs, Beaumont, TX) and the 5 bands were quantitated using scanning densitometry (Garrick *et al.*, 1975).

*Cytotoxicity assays.* Primary monolayer cultures of hepatocytes from male F344 rats 160–180 g wt were prepared as we have previously described (Carr & Laishes, 1981). After a 3 h attachment period using Leibowitz L-15 medium (GIBCO Labs, Grand Island, NY) and 10% calf serum, the medium was changed to fresh medium containing 10% calf serum and the cells were then incubated without (controls) or with various cytidine analogues at the indicated doses. After a 24 h incubation period *in vitro*, trypan blue was added to the tissue culture cells and the percentage of suvival was assessed. Cell survival was measured as the percentage viable attached cells in the experimental flasks compared to the percent viable attached cells in the control of flasks.

*Reagents.* Chemicals used were: 5-azacytidine and 5-aza-2′-deoxycytidine (Sigma Chemical Company, St Louis, MO); 6-azacytidine, 5-fluoro-2′-deoxycytidine and 5-fluorocytidine (Calbiochem); 5,6 dihydro-5-azacytidine and tetrahydrouridine were a gift from the Drug Synthesis and Chemistry Branch of the National Cancer Institute, NIH, Bethesda, MD. 5-fluorocytidine was synthesized by Calbiochem-Behring (San Diego, CA); pseudoisocytidine for use in tissue culture was a kind gift of Dr J.J. Fox, Laboratory of Organic Chemistry, Sloan-Kettering Institute for Cancer Research, Rye, New York.

### Results

*Carcinogenicity*

We investigated the carcinogenicity of 3 doses of 5-azacytidine, using 2.5, 0.25, and 0.025 mg kg$^{-1}$. The highest dose chosen was the lowest dose that appeared to be carcinogenic in a preliminary experiment (Carr *et al.*, 1984). A large excess of rats was used for the 5-azacytidine compared to the control regimen (Regimens 1 and 2, Table I) in anticipation of a steady attrition rate at this dose of 5-azacytidine. However, attrition was low and 87% survived. Table II shows that 18% of the rats treated with the highest dose of 5-azacytidine developed non-testicular tumours compared to none in the control group (Regimen 2, Table II). In this group, 6 of the 87 evaluable rats also had histological evidence of intraperitoneal fat necrosis. No rats developed tumours at the lower doses of 5-azacytidine (Regimens 3 and 4, Table II). While 20% of the control rats developed testicular tumours, particularly of the Leydig cell type, as has been reported elsewhere as a feature of aging (Goodman *et al.*, 1979), 3 times the incidence rate of

**Table I** Treatment regimens

| Regimen | Treatment type | Dose | Route | Frequency | Duration |
|---|---|---|---|---|---|
| 1. | Age controls: NaCl 0.9% | 0.5 ml | i.p. | 3 × weekly | 1 yr |
| 2. | 5-azacytidine | 2.5 mg kg$^{-1}$ | i.p. | 3 × weekly | 1 yr |
| 3. | 5-azacytidine | 0.25 mg kg$^{-1}$ | i.p. | 3 × weekly | 1 yr |
| 4. | 5-azacytidine | 0.025 mg kg$^{-1}$ | i.p. | 3 × weekly | 1 yr |
| 5. | 5-deoxyazacytidine + THU[b] 27.5 mg kg$^{-1}$ | 2.5 mg kg$^{-1}$ | i.p. | 3 × weekly | 1 yr |
| 6. | 5-fluorodeoxycytidine + THU 27.5 mg kg$^{-1}$ | 2.5 mg kg$^{-1}$ | i.p. | 3 × weekly | 1 yr |
| 7. | 5-fluorocytidine + THU 27.5 mg kg$^{-1}$ | 2.5 mg kg$^{-1}$ | i.p. | 3 × weekly | 1 yr |
| 8. | 5,6-dihydro-5-azacytidine | 50 mg kg$^{-1}$ | i.p. | 3 × weekly | 1 yr |
| 9. | 6-azacytidine | 2.5 mg kg$^{-1}$ | i.p. | 3 × weekly | 1 yr |
| 10. | 5-azacytidine | 2.5 mg kg$^{-1}$ | i.p. | 3 × weekly | 1 yr |
| | + cytidine | 25 mg kg$^{-1}$ | i.p. | 3 × weekly | 1 yr |
| 11. | 5-azacytidine at day 21 to timed pregnant rats | 10 mg kg$^{-1}$ | i.p. to mother | once | [a] |
| 12. | 5-azacytidine to 25 g weanling rats | 5 mg kg$^{-}$ | i.p. | once | [d] |
| 13. | PH/DEN[c] 30 mg kg$^{-1}$ | | i.p. | once | |
| | 5-azacytidine | 2.5 mg kg$^{-1}$ | i.p. | 3 × weekly | 1 yr |
| 14. | THU | 27.5 mg kg$^{-1}$ | i.p. | 3 × weekly | 1 yr |

[a]Offspring examined 1 yr after birth; [b]THU, Tetrahydrouridine; [c]PH, 2/3 Partial Hepatectomy; DEN, Diethylnitrosamine 18 h after PH; [d]Rats examined 1 yr after birth.

**Table II** Carcinogenicity study of 5-azacytidine and analogues in male F344 rats: Overall results

| Category treatment | No. rats | | No. rats with non-testis tumours (%) | P | No. rats with testis tumours (%) | P | % rats with any tumour | No. rats with Leydig cell hyperplasia |
|---|---|---|---|---|---|---|---|---|
| | Initial | Evaluable[a] | | | | | | |
| 1. Controls | 50 | 49 | 0 | | 10 (20) | | 20% | 6 |
| 2. 5-azacytidine 2.5 mg kg$^{-1}$ | 100 | 87 | 16 (18) | <0.01 | 56 (64) | <0.001 | 72% | 11 |
| 3. 5-azacytidine 0.25 mg kg$^{-1}$ | 10 | 10 | 0 | | 2 (20) | NS | 20% | |
| 4. 5-azacytidine 0.025 mg kg$^{-1}$ | 10 | 10 | 0 | | 1 (10) | NS | 10% | |
| 5. 5-deoxyazacytidine + THU | 10 | 10 | 0 | | 0 | | 0 | 2 |
| 6. 5-fluorodeoxycytidine + THU | 10 | 10 | 0 | | 1 (10) | NS | 10% | 2 |
| 7. 5-fluorocytidine + THU | 10 | 10 | 0 | | 0 | | 0 | 2 |
| 8. 5,6-dihydro-5-azacytidine | 10 | 9 | 1 (11) | 0.12 | 2 (22) | NS | 33% | 1 |
| 9. 6-azacytidine | 15 | 12 | 0 | | 2 (17) | NS | 17% | 9 |
| 10. 5-azacytidine + cytidine | 10 | 5 | 0 | | 2 (40) | NS | 40% | |
| 11. 5-azacytidine to 5 pregnant rats (offspring: 13 male, 9 female) | 22 | 22 | 3 (14) | 0.03 | 3/13 (23) | NS | 27% | 3/13 |
| 12. 5-azacytidine to weanlings | 10 | 9 | 1 (11) | NS | 2 (22) | NS | 33% | 6 |
| 13. PH/DEN→5-azacytidine | 10 | 8 | 5 (63) (8/8 hyperplastic liver nodules) | <0.001 | 1 (13) | NS | 75% | |
| 14. THU (controls) | 10 | 10 | 0 | | 3 (30) | NS | 30% | 5 |

PH, Partial hepatectomy; DEN, diethylnitrosamine 30 mg kg$^{-1}$ i.p. 18 h after PH; THU, tetrahydrouridine; NS, not significantly different from control values; *P* using Fisher's exact test; [a]Evaluable, no. of rats surviving till end of experiment.

testicular tumours were seen in the group treated with 2.5 mg kg$^{-1}$ 5-azacytidine (Regimen 2, Table II). The doses for the analogues were 10% of the $LD_{10}$ dose, obtained in preliminary experiments, except for 5,6-dihydro-5-azacytidine, for which no practicable $LD_{50}$ dose could be found. No excess of non-testicular tumours was seen with any of the analogues. In the 5,6-dihydro-5-azacytidine group, one rat developed a sarcoma but this was not statistically significant (Regimen 8, Tables II & III) and a single rat had intraperitoneal fat necrosis. It was interesting that in the group of rats treated with 5′-deoxyazacytidine (Regimen 5, Table II), all 10 developed testicular atrophy, characterized by reduction in size of seminiferous tubules secondary to absence of spermatogenesis, and the presence of oedema and focal interstitial fibrosis. Two of the 10 also showed Leydig cell hyperplasia. In a small group of rats treated with 5-azacytidine plus a 10-fold concentration of cytidine (Regimen 10, Table 2) no excess of nontesticular tumours were noted. In the group of rats that received a single initiating dose of the liver carcinogen DEN (Regimen 13, Table II), all the surviving rats developed hyperplastic liver nodules and a high incidence of non-hepatic, non-testicular primary tumours. Previous work has established that low single DEN doses after a two-thirds partial hepatectomy do not result in liver tumours without subsequent tumour promotion (Carr *et al.*, 1984; Pitot *et al.*, 1978). Tetrahydrouridine control rats did not develop any excess of non-testicular tumours nor did they show testicular atrophy (Regimen 14). In a test for transplacental carcinogenicity, timed pregnant rats were given a single large dose of 5-azacytidine on the last day of their pregnancy. Earlier periods were not used because of the known fetotoxicity of 5-azacytidine (Schmahl *et al.*, 1985 and our unpublished data). A high incidence of non-testicular primary tumours was noted (3 male, 1 female) but no excess of testicular tumours was found (Regimen 11, Table II). When 5-azacytidine was given as a single dose to weanling rats (Regimen 12, Table II), a single rat developed a non-testicular tumour.

*Spectrum of tumour types*

The sepctrum of primary tumour types that was induced in response to chronic administration for the various cytidine analogues is shown in Table III. Tumours were those of the lymphoid system, kidney, lung, skin at the site of injection, sarcomas and mesotheliomas. Hyperplastic liver nodules were found in the rats which were treated with 5-azacytidine after a single initiating dose of the hepatic carcinogen diethylnitrosamine (Regimen 13, Table III).

*Cytotoxicity of cytidine analogues*

Using a primary monolayer culture system of freshly isolated normal rat hepatocytes to test the cytocidal effects of the cytidine analogues, the cytotoxic actions of the drugs used in the carcinogenicity study were compared (Table IV). The analogues were incubated with or without the deaminase inhibitor tetrahydrouridine. The results are shown for incubations without tetrahydrouridine, but they were identical in the presence of tetrahydrouridine. The most toxic analogue was 5-azacytidine, followed by 5-fluorocytidine and 5,6-dihydro-5-azacytidine. In this assay, differences of less than 10% are not considered significant. The changes in rat weights over the duration of the experiment with the different analogues are shown in Figure 1.

*Minor haemoglobin induction by cytidine analogues.*

The ability of cytidine analogues to induce minor haemoglobin synthesis was investigated using 3 different mammalian species: sheep, rats and mice. Advantage was taken of the fact that in sheep and mice, the induction of an otherwise silent or minor haemoglobin can be unambiguously demonstrated. DBA/2J mice were chosen following the report that this inbred strain has a minor haemoglobin comprising ~20% of the total haemoglobin, is electrophoretically distinct, and increased synthesis is inducible by an anaemic stress (Alter *et al.*, 1982). Another species with a foetal haemoglobin switch which is capable of induction is the sheep (Young *et al.*, 1978). Figure 2 shows the patterns of haemoglobin electrophoresis in the 3 species, sheep, rats and mice. Chronic administration of $2.0\,mg\,kg^{-1}$ 5-azacytidine to 2 sheep for 4 months resulted in no induction of the minor haemoglobin (Figure 2c, d). However, rats in the carcinogenicity studies of this report, showed qualitative changes with the 5-azacytidine doses used (Figure 2e-g). An increase was found in the 2 anodic of the 5 total haemoglobin bands that were visualized on the cellulose acetate electrophoretograms and the disappearance of one band. However, no unambiguous rat haemoglobin changes were seen with any of the other analogues. The significance of the multiple rat haemoglobins is not clear, since they have not been characterized, to our knowledge. In the DBA/2J mice, a greater than 30% increase (Table V) in the minor haemoglobin component was seen in 8 of 9 mice treated with 5-azacytidine $1.6\,mg\,kg^{-1}$, in 6 of 10 mine treated with 5,6-dihydro-5-azacytidine $1.6\,mg\,kg^{-1}$, and in 2 of 5 mice treated with 5-aza-2′-deoxy-5-azacytidine $1.6\,mg\,kg^{-1}$. No significant increase in the minor haemoglobin was seen in the saline controls or the mice treated with 6-azacytidine.

Table VI shows the cytidine analogues that were used in this study, and compares their reported activity as inhibitors of methylation *in vitro* with their activity as complete carcinogens and inducers of haemoglobin synthesis. No clear relationship between the potency of the analogues as inhibitors of DNA methylation and their action as carcinogens is apparent.

### Discussion

The aims of this study were to investigate the carcinogenicity, toxicity and the haemoglobin activating properties of 5-azacytidine, and to initiate the evaluation of some related analogues to determine whether there was a safer and less toxic analogue that might retain the gene activating properties. The results of this study were severalfold: (1) we confirmed that 5-azacytidine is a complete carcinogen for several tissues and a probable tumour promoter for liver; (2) none of the other analogues tested were significantly carcinogenic, although the sample size was small; (3) a single dose of 5-azacytidine delivered transplacentally to neonates was carcinogenic; (4) several analogues induced minor haemoglobin synthesis in mice, keeping open the possibility that minor haemoglobin induction and carcinogenicity are not irrevocably linked; and (5) only 5-deoxyazacytidine caused dramatic testicular atrophy.

In the main carcinogenicity study of this report, in which 5-azacytidine $2.5\,mg\,kg^{-1}$ given over 12 months was compared to age-matched control rats (Regimens 1 and 2, Table II), we found that 5-azacytidine induced both a 3-fold increase in the incidence of testicular tumours over the spontaneous incidence which occurs in normal rats with age (Goodman *et al.*, 1979), and also a large increase in the incidence of non-testicular tumours. In addition, we were able to confirm our preliminary observation that 5-azacytidine appears to enhance the induction of hyperplastic (neoplastic) liver nodules in rats that were treated with a single initiating dose of diethylnitrosamine. Thus, it appears to be a tumour promoter in the liver. We did not find any significant carcinogenic activity of the cytidine analogues which we tested. The concomitant administration of cytidine with 5-azacytidine did not appear to suppress the increased

**Table III** Organ distribution of tumours

| *Regimens (No. rats)* | *Lymphoma* | *Renal* | *Lung* | *Skin* | *Mesothelioma* | *Sarcoma* | *Testis* | *Hyperplastic liver nodules* |
|---|---|---|---|---|---|---|---|---|
| 1. Controls (49) | | | | | | | 10 | |
| 2. 5-azacytidine $2.5\,mg\,kg^{-1}$ (87) | 4 | 4 | 1 | 3 | 2 | 2 | 56 | |
| 3. 5-azacytidine $0.25\,mg\,kg^{-1}$ (10) | | | | | | | 2 | |
| 4. 5-azacytidine $0.025\,mg\,kg^{-1}$ (10) | | | | | | | 1 | |
| 5. 5-deoxycytidine + THU (10) | | | | | | | | |
| 6. 5-fluorodeoxycytidine + THU (10) | | | | | | | 1 | |
| 7. 5-fluorocytidine + THU (10) | | | | | | | | |
| 8. 5,6-dihydro-5-azacytidine (9) | | | | | | 1 | 2 | |
| 9. 6-azacytidine (12) | | | | | | | 2 | |
| 10. 5-azacytidine + cytidine (5) | | | | | | | 2 | |
| 11. 5-azacytidine to 5 pregnant rats (offspring: 13 male, 9 female) | 1 | | 1 | 1 | | | 3 | 1 |
| 12. 5-azacytidine to weanlings (9) | 1 | | | | | | 2 | |
| 13. PH/DEN→5-azacytidine (8) | | | 2 | 3 | | | 1 | 8 |
| 14. THU | | | | | | | 3 | |

**Table IV** Cytotoxicity of cytidine analogues

| *Treatment*[a] | *% Survival*[b] |
|---|---|
| Tetrahydrouridine | 100 |
| Cytosine | 85±4 |
| Cytidine | 77±6 |
| Pseudoisocytidine | 70±7 |
| 5-fluorocytosine | 69±6 |
| 6-azacytidine | 67±5 |
| 5-aza-2′-deoxycytidine | 63±4 |
| 5-fluoro-2′-deoxycytidine | 62±8 |
| 5,6-dihydro-5-azacytidine | 50±7 |
| 5-fluorocytidine | 47±11 |
| 5-azacytidine | 32±8 |

[a]All drugs were incubated with freshly-isolated attached normal hepatocytes *in vitro* at $1\times10^{-4}$ M for 24 h. 5-azacytidine and its analogues were incubated with or without tetrahydrouridine $1\times10^{-3}$ M. Tetrahydrouridine alone was used at $1\times10^{-3}$ M; [b]% survival = % attached, viable cells in treatment flasks/attached viable cells in control flasks without test toxin, using trypan blue exclusion assay.

**Figure 1** Effects of chronic administration of cytidine analogues on rat weights. Rats were chronically administered cytidine analogues intraperitoneally in 0.9% NaCl solution (experiments) or NaCl solution only (controls). The regimens were as in **Table I**, and the rats were weighed monthly. The datum points are averages for all the rats in each regimen; controls (○); 5-azacytidine (■); 5-fluorocytidine + THU (●); 5-deoxyazacytidine + THU (△); 5-fluorodeoxycytidine + THU (□); 5,6-dihydro-5-azacytidine (▲).

number of testis tumours, although non-testicular tumours were not seen in a small series (Regimen 10, Table I). It was of great interest however, that 5-azacytidine induced transplacental carcinogenesis of non-testicular tumours (Regimen 11, Table I). In another report, 5-azacytidine was found to induce transplacental carcinogenesis in mice (Schmahl *et al.*, 1985).

The results of 5-azacytidine action are expected to be complex. On the one hand, 5-azacytidine is capable of enhancing or inducing the metastatic capacity of various tumour cell lines (Olsson & Forchammer, 1984; Trainer *et al.*, 1985; Ormerod *et al.*, 1986), activating silent retroviral genomes (Jaenisch *et al.*, 1985), enhancing the induction by various carcinogens of gamma-glutamyltransferase positive liver foci (Denda *et al.*, 1985), of altering cellular DNA which is incapable of inducing transformation (Venolia *et al.*, 1982), and of inducing tumorigenesis in various cells in culture (Venolia *et al.*, 1982; Harrison *et al.*, 1983; Benedict *et al.*, 1977; Marquardt & Marquardt, 1977), even though it does not appear to be a significant mutagen in mammalian cells (Landolph & Jones, 1982; Frost *et al.*, 1984; Momparler *et al.*, 1984; Delers *et al.*, 1984; Bouck *et al.*, 1984; Jones, 1984). On the other hand, 5-azacytidine has been shown to induce differentiation in both non-transformed as well as in neoplastic cells in culture (Constantinides *et al.*, 1977, 1978; Jones & Taylor, 1980; Walker *et al.*, 1984; Creusot *et al.*, 1982; Darmon *et al.*, 1984; Pinto *et al.*, 1984). 5-Azacytidine is a cell toxin and has been used to treat leukaemia (von Hoff *et al.*, 1976) and can induce the expression of new cell surface antigens and thus increase the effectiveness of immunosurveillance in immunocompetent animals (Frost *et al.*, 1984). Thus, there appear to be opposite sets of biological actions with respect to carcinogenicity. The explanation may reside in the net effect of the genes that are activated by 5-azacytidine under a given set of experimental conditions. We did not find any evidence of carcinogenicity for 5-azacytidine at log doses below 2.5 mg $kg^{-1}$ in rats. The spectrum of tumour types were similar to that seen previously (Carr *et al.*, 1984; Schmahl *et al.*, 1985); *viz.*, tumours of the lymphoid system, skin, lung and kidney, as well as sarcomas and mesotheliomas (Table III). The skin tumours occurred only at the site of injection, suggesting that there was no need for further drug processing for the carcinogenic action to occur.

Two assays for toxicity were used in our experiments. The first was the change in rat weights with time during the carcinogenicity studies (Figure 1). Very few rats died during the experiment and most weight loss was found for the rats treated with the fluorinated cytidine derivatives and with 5-deoxyazacytidine. The second assay was a 24 h cytotoxicity study using primary monolayer cultures of freshly prepared normal hepatocytes (Table V). The 3 drugs with most cytotoxic effects were 5-azacytidine, 5-fluorocytidine and 5,6-dihydro-5-azacytidine. The addition of tetrahydrouridine to the analogues in cell culture did not influence the toxicity.

The electrophoretic pattern of haemoglobin components of rats was quite complicated and 5 major haemoglobins were visualized, with several minor bands appearing on the densitometry tracings of the electrophoretograms. The changes included an increase in density in the 2 anodic bands and an almost complete disappearance of a third haemoglobin band (Figure 2e-g). As a result of the complexities inherent in interpreting the multiple haemoglobin bands in rat blood, 2 other species with well-studied and less complicated haemoglobin bands were used. No minor haemoglobin component induction was found in the 2 sheep that were used. However, DBA/2J female mice which have a major and a minor haemoglobin, showed an increased synthesis of the minor haemoglobin at both high and low doses of 5-azacytidine. This increased minor haemoglobin synthesis in mice was also induced by 5,6-dihydro-5-azacytidine and 5-aza-2′-deoxycytidine (Table V). Interestingly, increased minor haemoglobin synthesis did not take place within the first few days of cytidine analogue treatment of the mice, but appeared slowly over a 2 month period. Similarly, 5,6-dihydro-azacytidine induces an increase in foetal haemoglobin in humans (Carr *et al.*, 1987). The mechanism by which cytidine analogues alter minor haemoglobin synthesis is not entirely clear. Although gene activation through DNA methylation changes is a candidate mechanism, other possibilities exist, such as erythroid pool stress. Although we did not observe haematocrit decreases of more than 20%, subtle erythroid stress cannot be excluded.

On comparing the results for minor haemoglobin induction (Table IV) with the carcinogenicity results (Table II), it appeared that 5-azacytidine was able to induce an increased minor haemoglobin synthesis in mice and altered haemoglobin synthesis in rats at lower doses than were required for the induction of rat tumours. However, it cannot be assumed that mouse and rat tumorigenic doses will be the same, and separate carcinogenicity studies will be required for the mouse. Two analogues appeared to have haemoglobin activating properties similar to those observed

**Figure 2** Electrophoretic separation of haemoglobins of mice, sheep and rats treated with or without 5-azacytidine. Lanes a and b, cellulose acetate electrophoresis at pH 8.6 of peripheral blood haemoglobins from DBA/J female mice treated (a) with or (b) without 5-azacytidine $1.6\,mg\,kg^{-1}$ for 60 days. The horizontal arrow by lane a indicates the induced band. Lanes c and d, cellulose acetate electrophoresis at pH 8.6 of sheep blood haemoglobins; (c) pretreatment; (d) after 4 months of 5-azacytidine $2.0\,mg\,kg^{-1}$ treatments. Lanes e, f, and g, cellulose acetate electrophoresis at pH 8.6 of peripheral blood haemoglobins from male F344 rats: (e) normal controls, (f, g), 5-azacytidine ($2.5\,mg\,kg^{-1}$) treated rats after 60 days. The vertical arrow shows the direction of electrophoresis; ○, indicates the origin, to show anodic and cathodic migration of rat haemoglobin bands. The 4 horizontal arrows by lane e indicate, in descending order: two normal cathodic bands which are increased by 5-azacytidine; precipitated haemoglobin at the origin (heavy band above plane of the gel); the normal anodic band which decreases with 5-azacytidine treatment.

**Table V** Effects of cytidine analogues on minor haemoglobin synthesis in DBA/2J mice

| | *Minor Hb as % total Hb (mean ± s.d.)* | | | |
|---|---|---|---|---|
| *Analogue* | *Initial* | *30 days* | *60 days* | *% increase over control minor Hb at 60 days* |
| Controls ($n=8$) | 14.8±0.6 | 15.3±2.0 | 16.3±1.8 | – |
| 6-azacytidine ($n=5$) | 14.3±0.7 | 14.9±1.4 | 15.6±1.5 | – |
| 5-azacytidine $16\,mg\,kg^{-1}$ ($n=10$) | 14.5±1.5 | 17.0±1.9 | 22.2±4.2 | 36 |
| 5-azacytidine $1.6\,mg\,kg^{-1}$ ($n=9$) | 15.6±1.3 | 15.3±5.8 | 21.7±3.8 | 33 |
| 5,6-dihydro-5-azacytidine $1.6\,mg\,kg^{-1}$ ($n=10$) | 14.2±3.6 | 25.0±3.1 | 24.7±1.8 | 52 |
| 5-aza-2′-deoxycytidine $1.6\,mg\,kg^{-1}$ ($n=5$) | 14.3±1.4 | 16.4±2.4 | 21.3±0.7 | 31 |

**Table VI** Inhibition of DNA methylation and carcinogenesis

| *Analogue* | *Inhibition of methylation*[a] | *Carcinogenic activity*[b] | *Mouse minor Hb synthesis*[c] | *Human foetal Hb synthesis* |
|---|---|---|---|---|
| 6-azacytidine | – | – | – | ND |
| 5-azacytidine | ++ | +++ | + | ++[d] |
| 5-aza-2′-deoxyazacytidine | +++ | – | + | ND |
| 5-fluorocytidine | ND | – | ND | ND |
| 5-fluorodeoxycytidine | ++ | – | ND | ND |
| 5,6-dihydro-5-azacytidine | – | ± | + | +[e] |

[a]*In vitro* data (abstracted from Jones & Taylor, 1980, Table I); [b]This paper, **Table II**; [c]This paper, **Table IV**; [d]Ley *et al.* (1982); Charache *et al.* (1983); [e]Carr *et al.* (1987); ND Not done.

for 5-azacytidine, *viz.*, 5,6-dihydro-5-azacytidine and 5-aza-2′-deoxycytidine. These two analogues had no significant carcinogenic activity in our study. 5-aza-2′-deoxycytidine appears particularly promising in this respect, since it was of only moderate toxicity (Table V and Figure 1) and did not induce tumours in a small carcinogenicity series, yet retained gene activating properties. These results confirm that 5-azacytidine is a complete carcinogen in the male F344 rat, and show that it has transplacental carcinogenic properties, and can act as a hepatic tumour promoter. No 5-azacytidine-induced tumorigenicity was seen below $2.5\,mg\,kg^{-1}$ in our study. 5-Azacytidine was able to alter haemoglobin synthesis in rats and mice. Five other cytidine analogues were also tested for carcinogenicity in experiments with low numbers of rats, in which tumorigenicity was not clearly demonstrable. However, 2 of these apparently non-carcinogenic analogues, 5,6-dihydro-5-azacytidine and 5-aza-2′-deoxycytidine retained the ability to increase minor haemoglobin synthesis. These results suggest that it may be possible to separate the carcinogenic from the potentially useful gene-activating properties in some cytidine analogues. From the results reported here and elsewhere (Table VI) there does not appear to be a clear relationship among cytidine analogues between potency as inhibitors of DNA methylation and carcinogenic activity.

### References

ALTER, B.P., CAMPBELL, A.S., HOLLAND, J.G. & FRIEND, C. (1982). Increased in the mouse minor haemoglobin during erythroid stress: A model for haemoglobin regulation. *Exptl. Hematol.*, **10**, 754.

BENEDICT, W.F., BANERJEE, A., GARDNER, A. & JONES, P.P. (1977). Induction of morphological transformation in mouse CDH/10T$\frac{1}{2}$ clone 8 cells and chromosomal damage enhanced A ($T_1$) C 1-3 cells by cancer chemotherapeutic agents. *Cancer Res.*, **37**, 2202.

BOEHM, T.L.J. & DRAHOVSKY, D. (1979). Effect of carcinogen ethionine on enzymatic methylation of DNA sequences with various degrees of repetitiveness. *Eur. J. Cancer*, **15**, 1167.

BOEHM, T.L.J. & DRAHOVSKY, D. (1981). Hypomethylation of DNA in raji cells after treatment with N-methyl-N-nitrosourea. *Carcinogenesis*, **2**, 39.

BOUCK, N., KOKKINAKIS, D. & OSTROWSKY, J. (1984). Induction of a step in carcinogenesis that is normally associated with mutagenesis by non-mutagenic concentrations of 5-azacytidine. *Mol. Cell. Biol.*, **4**, 1231.

CARR, B.I., REILLY, J., SMITH, S.S., WINBERG, C. & RIGGS, A. (1984). The tumorigenicity of 5-azacytidine in the male Fischer rat. *Carcinogenesis*, **5**, 1583.

CARR, B.I. & LAISHES, B.A. (1981). Carcinogen-induced drug resistance in rat hepatocytes. *Cancer Res.*, **41**, 1715.

CARR, B.I., RAHBAR, S., DOROSHOW, J.H. & 4 others (1987). Fetal hemoglobin gene activation in a phase II study of 5,6-dihydro-5-azacytidine for bronchogenic carcinoma. *Cancer Res.*, **47**, 4199.

CHABNER, B.A., DRAKE, J.C. & JOHNS, D.J. (1973), Deamination of 5-azacytidine by a human leukemia cell cytidine deaminase. *Biochem. Pharmacol.*, **22**, 2763.

CHARACHE, S., DOVER, G., SMITH, K., TALBOT, JR., C.C., MOYER, M. & BOYER, S. (1983), Treatment of sickle cell anemia with 5-azacytidine results in increased fetal hemoglobin production and is associated with non-random hypomethylation of DNA around a gamma-delta-beta-globin gene complex. *Proc. Natl Acad. Sci. USA*, **80**, 4842.

CHEAH, M.S.C., WALLACE, C.D. & HOFFMAN, R.N. (1984). Hypomethylation of DNA in human cancer cells: A site-specific change in c-myc oncogene. *J. Natl Cancer Onst.*, **73**, 1057.

CIHAK, A. (1974). Biological effect of 5-azacytidine in eurokaryotes. *Oncology*, **30**, 405.

CIHAK, A. & VESELY, J. (1969). Altered liver regeneration in partially hepatectomized rats following 5-azacytidine treatment. *Collect. Czech. Chem. Commun.*, **34**, 910.

CONSTANTINIDES, P.G., JONES, P.A. & GEVERS, W. (1977). Functional striated muscle cells from non-myoblast precursors following 5-azacytidine treatment. *Nature*, **267**, 364.

CONSTANTINIDES, P.G., TAYLOR, S.M. & JONES, P.A. (1978). Phenotypic conversion of cultured mouse embryo cells by azapyrimidine nucleosides. *Devel. Biol.*, **66**, 57.

CREUSOT, F., ACS, G. & CHRISTMAN, J.K. (1982). Inhibitional DNA methyltransferase and induction of Friend Erythroleukemia cell differentiation by 5-azacytidine and 5-aza-2′-deoxycytidine. *J. Biol. Chem.*, **257**, 2041.

DARMON, N., NICOLAS, J.-F. & LAMBLIN, D. (1984). 5-azacytidine is able to induce the conversion of teratocarcinoma-derived mesenchymal cells into epithelial cells. *EMBO, J.*, **3**, 961.

DELERS, A., SZPIRER, J., SZPIRER, C. & SAGGIORIO, D. (1984). Spontaneous and 5-azacytidine induced re-expression of ornithine carbamoyl transferase in hepatoma cells. *Mol. Cell. Biol.*, **4**, 804.

DENDA, A., RAO, P.M., RAJALAKASHMI, S. & SARMA, D.S.R. (1985). 5-azacytidine potentiates initiation induced by carcinogens in rat liver. *Carcinogenesis*, **6**, 145.

DOEFLER, W. (1983). DNA methylation and gene activity. *Ann. Rev. Biochem.*, **52**, 93.

FEINBERG, A.P. & VOGELSTEIN, B. (1983*a*). Hypomethylation distinguishes genes of some human cancers from their normal counterparts. *Nature*, **301**, 89.

FEINBERG, A.P. & VOGELSTEIN, B. (1983*b*). Hypomethylation of ras oncogenes in primary human cancers. *Biochem. Biophys. Res. Commun.*, **111**, 47.

FROST, P., LITEPLO, R.G., DONAGHUE, T.P. & KERBEL, R.S. (1984). Selection of strongly immunogenic T $um^-$ variance from tumors at high frequency using 5-azacytidine. *J. Exp. Med.*, **159**, 1491.

GAMA-SOSA, M.A., SLAGEL, V.A., TREWYN, R.W. & 4 others (1983). The 5-methylcytosine content of DNA from human tumors. *Nucleic Acids Res.*, **11**, 6883.

GARRICK, L.M., SHARMA, V.S., McDONALD, M.J. & RANNEY, H.M. (1975). Rat hemoglobin heterogeneity. Two structurally distinct – chains and functional behavior of selected components. *Biochem. J.*, **149**, 245.

GOELZ, S.E., VOGELSTEIN, B., HAMILTON, S.R. & FEINBERG, A.P. (1985). DNA from benign and malignant human colon neoplasms is hypomethylated. *Science*, **228**, 187.

GOODMAN, D.A., WARD, J.M., SQUIRE, R.A., CHU, K.C. & LINHART, M.S. (1979). Neoplastic and non-neoplastic lesions in aging F344 rats. *Toxicol. Appl. Pharmacol.*, **48**, 237.

HARRISON, J.J., ANISOWICZ, A., GADI, I.K., RAFFELD, M. & SHER, R. (1983). Azacytidine-induced tumorigenesis of CHBF/18 cells: correlated DNA methylation and chromosome changes. *Proc. Natl Acad. Sci. USA*, **80**, 6606.

HOLLIDAY, R. (1979). A new theory of carcinogenesis. *Br. J. Cancer*, **14**, 513.

HRODEK, O. & VESELY, J. (1971). 5-azacytidine in childhood leukemia. *Neoplasia*, **18**, 493.

JAENISCH, R., SCHNIEKE, A. & HARBERS, K. (1985). Treatment of mice with 5-azacytidine efficiently activates silent retroviral genomes in different tissues. *Proc. Natl Acad. Sci. USA*, **82**, 1451.

JONES, P.A. (1984). Gene activation by 5-azacytidine. In *DNA Methylation* Razin *et al.* (eds), p. 165. Springer-Verlag: New York.

JONES, P.A. (1986). DNA methylation in cancer. *Cancer Res.*, **46**, 461.

JONES, P.A. & TAYLOR, S.M. (1980). Cellular differentiation, cytidine analogs and DNA methylation. *Cell*, **20**, 85.

LANDOLPH, J.R. & JONES, P.A. (1982). Mutagenicity of 5-azacytidine and related nucleosides in C3H/10T$\frac{1}{2}$ clone and V79 cells. *Cancer Res.*, **42**, 817.

LAPEYRE, J.N., WALKER, M.S. & BECKER, F.F. (1981). DNA methylation and methylase level in normal and malignant mouse hepatic tissues. *Carcinogenesis*, **2**, 873.

LEY, T.J., DESIMONE, J., ANAGNOU, N.P. & 6 others (1982). 5-azacytidine selectively increases gamma-globin synthesis in a patient with beta + thalassemia. *New Engl. J. Med.*, **307**, 1469.

MARQUARDT, H. & MARQUARDT, H. (1977). Induction of molecular transformation and mutagenesis in cell cultures by cancer chemotherapeutic agents. *Cancer*, **40**, 1930.

MOMPARLER, R.L., SAMPSON, J., MOMPARLER, L.F. & RIVARD, G.E. (1984). Cell cycle effect and cellular pharmacology of 5-azacy-2-deoxycytidine. *Cancer Chemother. Pharmacol.*, **13**, 191.

NATIONAL CANCER INSTITUTE (1978). Bioassay of 5-azacytidine for possible carcinogenicity. Technical report, Series No. 42, CAS No. 320-67-2.

NEIL, G.L., MOXLEY, T.E. & MANAK, R.C. (1970). Enhancement by tetrahydrouridine of 1-beta-deca-arabinofuranosyl-cytosine (cytarabine) or activity in L1210 leukemic mice. *Cancer Res.*, **30**, 2166.

NYCE, J.W., WEINHOUSE, S. & MAGEE, P.N. (1983). 5-methylcytosine depletion during tumor development: An extension of the miscoding concept. *Br. J. Cancer*, **48**, 463.

OLSSON, L. & FORCHAMMER, J. (1984). Induction of the metastatic phenotype in a mouse-tumor model by 5-azacytidine, and characterization of an antigen associated with metastatic activity. *Proc. Natl Acad. Sci. USA*, **81**, 3389.

ORMEROD, E.J., EVERETT, C.A. & HART, I.R. (1986). Enhanced experimental metastatic capacity of a human tumor line following treatment with 5-azacytidine. *Cancer Res.*, **46**, 884.

PINTO, A., ATTADIA, V., FUSCO, A., FERRARA, F., SPADA, O.A. & DITA FIORE, P.P. (1984). 5-aza-2-deoxycytidine induces terminal differentiation of leukemic blasts from patients with acute myeloid leukemia. *Blood*, **64**, 922.

PISKALA, A. & SORM, F. (1964). Nucleic acids components and analogs. 1. Synthesis of 1-glycosyl derivatives of 5-azauracil and 5-azacytidine. *Collect. Czech. Chem. Commun.*, **29**, 2060.

PITOT, H.C., BARSNESS, L., GOLDSWORTHY, T. & KITAGAWA, T. (1978). Biochemical characterization of stages of hepato-carcinogenesis after a single dose of diethylnitrosamine. *Nature*, **271**, 456.

RAZIN, A., CEDAR, H. & RIGGS, A.D. (ed) (1984). *DNA Methylation: Biochemistry and Biological Significance*. Springer-Verlag: New York.

RAZIN, A. & RIGGS, A.D. (1980). DNA methylation and gene function. *Science*, **210**, 604.

REICHMAN, M. & PENMAN, S. (1973). Mechanism of inhibition of presynthesis by 5-azacytidine in HeLa cells. *Biochim. Biophys. Acta*, **84**, 282.

RIGGS, A.D. & JONES, P.A. (1983). 5-methylcytosine, gene regulation and cancer. *Adv. Cancer Res.*, **40**, 1.

SALAS, C.E., PFOHL-LESKOWICZ, A., LANG, M.C. & DIRHEIMER, G. (1979). Effect of modification by N-acetoxy-N-2-acetylamino-fluorene on the level of DNA methylation. *Nature*, **278**, 71.

SCHMAHL, W., GEBER, E. & LEHMACHER, W. (1985). Diaplacental carcinogenic effects of 5-azacytidine in NMRI mice. *Cancer Lett.*, **27**, 81.

STEWART, H.L., SNELL, K.C. & DUNHAM, L.J. (1980). Histological typing of liver tumors of the rat. *J. Natl Cancer Inst.*, **64**, 179.

STEWART, H.L., SNELL, K.C., DUNHAM, L.J. & SCHLYEN, S.M. (1959). Transplantable and transmissible tumors of animals. *Atlas of Tumor Pathology*, Section 12, Fascicle 40, AFIP.

STONER, G.D., SHIMKIN, M.B., KNIAZEFF, A.J., WEISBURGER, J.H., WEISBURGER, E.K. & GIORI, G.B. (1973). Test for carcinogenicity of food additives and chemotherapeutic agents by the pulmonary tumor response in strain A mice. *Cancer Res.*, **33**, 3069.

TRAINER, D.L., KLINE, T., MALLON, F., GREIG, R. & POSTE, G. (1985). Effect of 5-azacytidine on DNA methylation and of the malignant properties of B16 melanoma cells. *Cancer Res.*, **45**, 6124.

VENOLIA, L., GARTLER, S.M., WASSMAN, E.R., YEN, P., MOHANDAS, T. & SHAPIRO, L.J. (1982). Transformation with the DNA from 5-azacytidine-reactivated X chromosomes. *Proc. Natl Acad. Sci. USA*, **79**, 2352.

VESELY, J. & CIHAK, A. (1973). High frequency induction *in vivo* of mouse leukemia in AKR strain by 5-azacytidine and 5-iodo-2'-deoxyuridine. *Experientia*, **29**, 1132.

von HOFF, D.D., SLAVIK, M. & MUGGIA, F.M. (1976). 5-azacytidine: A new anticancer drug with effectiveness in acute myelogenous leukemia. *Ann. Int. Med.*, **85**, 237.

WALKER, C., RANNEY, D.F. & SHAY, J.W. (1984). 5-azacytidine-induced uncoupling of differentiation and tumorigenicity in a murine cell line. *J. Natl Cancer Inst.*, **73**, 877.

WEINTRAUB, H., LARSEN, A. & GROUDINE, M. (1981). Alpha-globin-gene switching during the development of chicken embryos: Expression and chromosome structure. *Cell*, **24**, 333.

WEISS, A.J., STAMBAUGH, J.E., MASTRANGELO, M.J., LAUCIUS, J.F. & BELLET, R.E. (1972). Phase I study of 5-azacytidine. *Cancer Chemother. Rep.*, **56**, 413.

WILSON, V.L. & JONES, P.A. (1983). Inhibitional DNA methylation by chemical carcinogen *in vitro*. *Cell*, **32**, 239.

YOUNG, N.S., BENZ, JR., E.J., KANTOR, J.A., KRETSCHMER, P. & NIENHUIS, A.W. (1978). Hemoglobin switching in sheep: Only the gamma-gene is in the active conformation in fetal liver but all the beta and gamma genes are in the active conformation in bone marrow. *Proc. Natl Acad. Sci. USA*, **75**, 5884.